# Epidemiological and molecular characterization of recombinant clade 2.5 lumpy skin disease virus in beef cattle across Thailand

**DOI:** 10.14202/vetworld.2026.4099-4114

**Published:** 2026-09-18

**Authors:** Phurida Sripipattanakul, Eaint Min Phyu, Chanakarn Nasamran, Supassama Chaiyawong, Bopit Puyati, Kamonpan Charoenkul, Alongkorn Amonsin

**Affiliations:** 1 Department of Veterinary Public Health, Faculty of Veterinary Science, Chulalongkorn University, Bangkok 10330, Thailand.; 2 National Institute of Animal Health, Department of Livestock Development, Bangkok 10900, Thailand.; 3 Center of Excellence for Emerging and Re-Emerging Infectious Diseases in Animals, Faculty of Veterinary Science, Chulalongkorn University, Bangkok 10330, Thailand.

**Keywords:** beef cattle, epidemiology, lumpy skin disease, molecular characterization, phylogenetic analysis, recombinant clade 2.5, Thailand, vaccination

## Abstract

**Background and Aim:**

Lumpy skin disease (LSD), caused by lumpy skin disease virus (LSDV), is an economically important transboundary cattle disease that emerged in Thailand in 2021. Despite its rapid nationwide spread, comprehensive epidemiological and molecular information on subsequent outbreaks remains limited. This study investigated LSDV occurrence and associated animal-level factors in Thai beef cattle during the 2022 outbreaks and characterized circulating viruses using molecular and phylogenetic analyses.

**Materials and Methods:**

A cross-sectional study was conducted from July to December 2022 on 208 beef cattle farms across 27 provinces of Thailand. In total, 482 cattle, including clinically affected and asymptomatic animals, were examined, and 523 samples comprising 387 hair and 136 skin lesion samples were collected. LSDV was detected by polymerase chain reaction targeting the *RPO30* gene. Associations between LSDV positivity and epidemiological factors were evaluated using logistic regression. Seventeen representative LSDV-positive samples were selected for *RPO30* and *GPCR* gene sequencing and phylogenetic analysis.

**Results:**

Overall, 358/482 (74.27%) cattle were LSDV-positive, including 304/405 (75.06%) clinically affected and 54/77 (70.13%) asymptomatic cattle. Weaners and calves had higher odds of LSDV positivity than adults, with adjusted odds ratio (aOR) of 1.89 (95% confidence interval [CI]: 1.02–3.69; p = 0.049) and 1.73 (95% CI: 1.01–3.01; p = 0.048), respectively. Vaccinated cattle had lower odds of positivity than unvaccinated cattle (aOR = 0.62; 95% CI: 0.39–0.98; p = 0.041). Skin lesion samples showed higher positivity (91.91%) than hair samples (69.77%). All 17 characterized Thai LSDVs showed 100% nucleotide identity within the *RPO30* and *GPCR* genes and clustered within recombinant clade 2.5, closely related to strains circulating in China and Southeast Asia. A characteristic 12-bp insertion was identified in the *GPCR* gene.

**Conclusion:**

The high LSDV positivity in both clinically affected and asymptomatic cattle demonstrates widespread viral circulation during the 2022 outbreaks. Younger age was associated with increased positivity, whereas vaccination was protective. The genetic uniformity and clade 2.5 clustering support regional circulation of closely related recombinant LSDVs. These findings emphasize sustained vaccination, molecular surveillance, animal movement control, and regional cooperation for effective LSD prevention and control.

## INTRODUCTION

Lumpy skin disease (LSD) is a systemic viral disease of cattle caused by lumpy skin disease virus (LSDV), a member of the genus *Capripoxvirus* within the family *Poxviridae* [1]. LSD is classified as a notifiable disease by the World Organization for Animal Health (WOAH) and represents a major threat to cattle health and productivity, resulting in substantial economic losses associated with reduced meat and milk production and disruptions to animal trade [2, 3]. LSDV can infect cattle of all ages, sexes, and breeds; however, disease severity tends to be greater in young, lactating, and immunocompromised animals [4]. Clinical manifestations commonly include fever, cutaneous edema, enlargement of superficial lymph nodes, and characteristic nodular skin lesions [3, 5]. Subclinical infection is also epidemiologically important because infected cattle without apparent clinical signs may contribute to virus circulation within and between farms [6]. LSDV is transmitted predominantly by blood-feeding arthropods, including stable flies and midges, although transmission through direct contact, contaminated fomites, and intrauterine routes has also been reported [7, 8].

LSD was first reported in Zambia in 1929 and subsequently became endemic in Africa before spreading to the Middle East, Europe, and Asia [2, 4]. In 2017, a recombinant vaccine-like LSDV associated with increased virulence was reported in Russia [9]. By 2019, LSD outbreaks had been reported in several Asian countries, including Bangladesh, China, and India, followed by further spread throughout Southeast Asia during 2020–2022, affecting Cambodia, Indonesia, Laos, Malaysia, Myanmar, Thailand, and Vietnam. In Thailand, LSD was first detected in March 2021 in Roi Et Province in the northeastern region and subsequently spread rapidly throughout the country [10]. By August 2022, the Bureau of Disease Control and Veterinary Services (BDCVS), Department of Livestock Development (DLD), Thailand, had reported LSD in 69 provinces, affecting more than 630,000 cattle on 280,000 farms, with cumulative morbidity and mortality rates of 34.09% and 3.60%, respectively [10]. Beef cattle were the livestock group most affected by LSD in Thailand, followed by dairy cattle and buffaloes [11]. During 2023–2024, the geographic distribution of LSD expanded further into East Asia, with outbreaks reported in South Korea in 2023 [12] and Japan in 2024 [13]. The continuing emergence and geographic spread of recombinant vaccine-like LSDV strains therefore represent important challenges to livestock production, disease prevention and control, and food security in Southeast Asia, including Thailand, an important regional beef producer.

Despite the rapid nationwide spread of LSD following its emergence in Thailand in 2021, comprehensive information integrating the epidemiological characteristics of infection with the molecular characteristics of circulating LSDV strains remains limited. Previous investigations have primarily addressed the early phase of the Thai outbreaks or focused on geographically restricted areas [10, 11]. Consequently, the distribution of LSDV positivity across multiple cattle-producing regions, the animal-level factors associated with infection, and the molecular relationships among viruses circulating in geographically separated areas of Thailand remain insufficiently characterized. This information is particularly important because the regional emergence of recombinant LSDV strains raises questions regarding the genetic characteristics and phylogenetic relationships of Thai LSDVs with strains circulating elsewhere in Asia. Furthermore, simultaneous epidemiological and molecular investigation is required to determine whether viruses detected across geographically diverse cattle populations belong to a common circulating lineage and to provide evidence relevant to surveillance and disease control strategies.

To address these knowledge gaps, a broad cross-sectional investigation encompassing beef cattle farms in 27 provinces across Thailand was conducted during the 2022 LSD outbreaks. The study integrated epidemiological assessment of animal-level factors with molecular detection and phylogenetic characterization of LSDV based on the *RPO30* and *GPCR* genes. This integrated approach enabled evaluation of LSDV infection across multiple geographic regions while providing molecular evidence regarding the genetic relatedness and phylogenetic placement of circulating Thai LSDVs. Such information is needed to improve understanding of LSDV epidemiology in Thailand and to provide a molecular basis for evaluating its relationship with recombinant strains circulating in East and Southeast Asia.

This study aimed to investigate the epidemiological and molecular characteristics of LSDV circulating in beef cattle during the 2022 outbreaks in Thailand. Specifically, the study aimed to determine LSDV positivity among sampled beef cattle; evaluate associations between LSDV positivity and animal-level factors, including age, sex, clinical status, and vaccination history; characterize representative LSDV-positive samples through sequencing of the *RPO30* and *GPCR* genes; and determine the genetic relationships and phylogenetic placement of Thai LSDVs relative to reference strains from different geographic regions and clades. The findings were intended to provide epidemiological and molecular evidence to support LSD surveillance, vaccination strategies, animal movement management, and future disease control programs in Thailand.

## MATERIALS AND METHODS

### Ethical approval

The study protocol was reviewed and approved by the Chulalongkorn University Animal Care and Use Committee (CU-VET IACUC; protocol no. 2031050, 01-09-2021). All animal-related procedures were conducted in accordance with institutional animal welfare requirements and the ARRIVE 2.0 guidelines. Hair and skin lesion samples, including samples from nodules, fistulas, sit-fasts, scabs, and scars, were collected by licensed veterinarians or appropriately trained personnel using procedures intended to minimize unnecessary discomfort to the animals. Before enrollment and sample collection, the study objectives, sampling procedures, and measures for maintaining data confidentiality were explained to the participating cattle farmers. Verbal informed consent was obtained from all participating cattle owners in accordance with the requirements of CU-VET IACUC. Personal protective equipment was used during field sampling to protect personnel and animals, and all data collected during the study were anonymized to protect the identities of participating farmers and animals.

### Study period and location

The cross-sectional study was conducted from July to December 2022 on 208 beef cattle farms across 27 provinces representing all regions of Thailand. The study areas comprised Chiang Rai, Phetchabun, Phitsanulok, and Sukhothai in northern Thailand; Ang Thong, Bangkok, Lopburi, Nonthaburi, Phra Nakhon Si Ayutthaya, Saraburi, and Suphanburi in central Thailand; Buri Ram, Chaiyaphum, Khon Kaen, Mukdahan, Roi Et, and Ubon Ratchathani in northeastern Thailand; Sa Kaeo in eastern Thailand; Kanchanaburi, Phetchaburi, Prachuap Khiri Khan, Ratchaburi, and Tak in western Thailand; and Chumphon, Narathiwat, Pattani, and Trang in southern Thailand (Figure 1). The provinces were selected purposively and included areas where LSD outbreaks in beef cattle had previously been reported. The selected provinces were also associated with beef cattle supply chains, including areas adjacent to international cattle movement routes and areas participating in the project to promote beef cattle production throughout the value chain. Participation was coordinated through the DLD network, with the cooperation of provincial livestock personnel [14].

**Figure 1:**
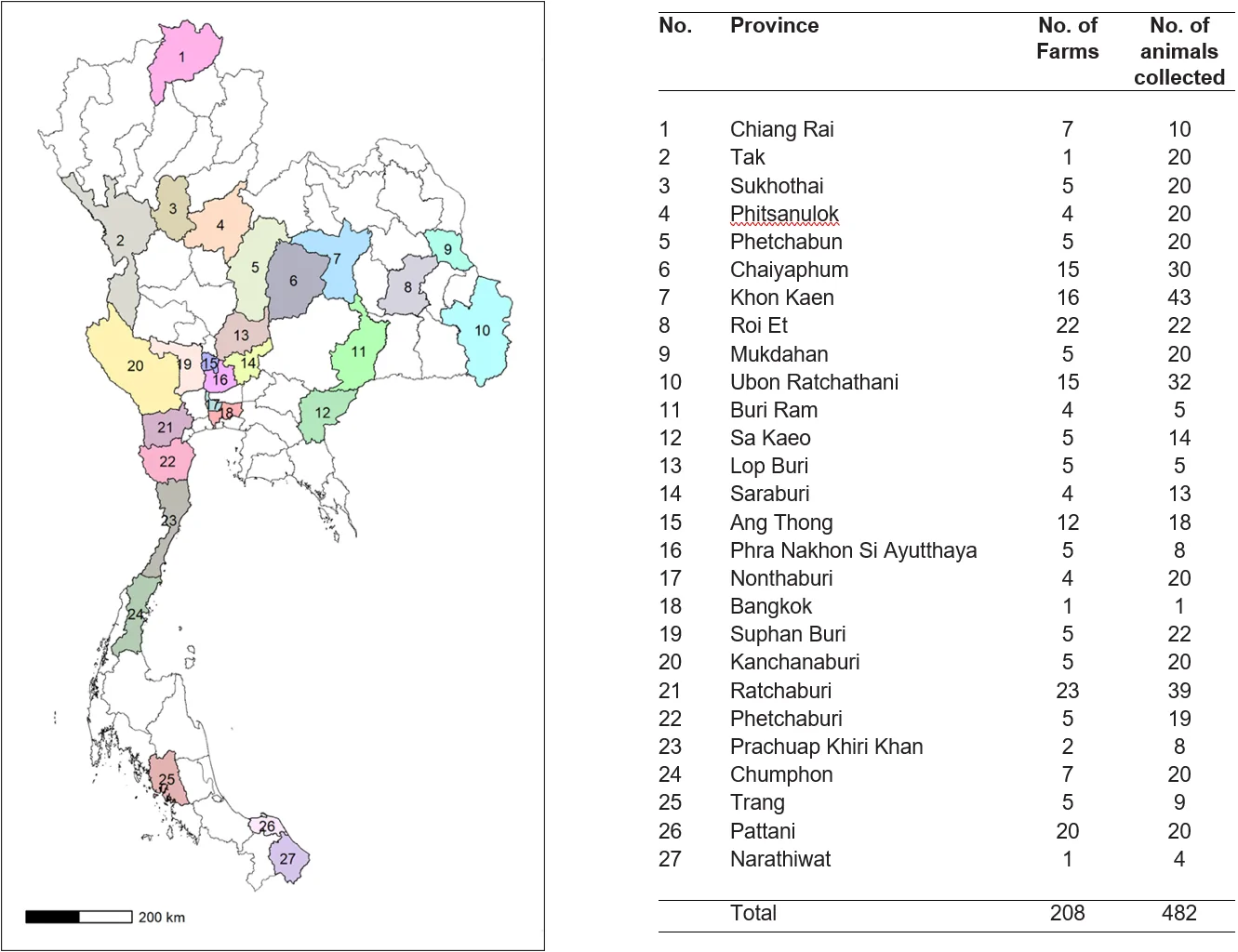
Geographic distribution of the 27 provinces in Thailand included in the study and province locations [Source: GADM.org (Https://gadm.org)].

### Study design and sample collection

A purposive cross-sectional study design was used to investigate LSDV infection in beef cattle and characterize circulating viral strains. The study integrated epidemiological data collection, conventional polymerase chain reaction (PCR) detection, and Sanger sequencing of the *RPO30* and *GPCR* genes. The minimum sample size was calculated using the single-proportion sample size function for apparent prevalence in EpiTools (Ausvet, Australia). The calculation assumed an expected prevalence of 10%, based on a previous study [15], a precision of 5%, a 99% confidence level, and an estimated beef cattle population of 9,756,642 animals in Thailand. The resulting minimum required sample size was 239 cattle; however, 482 cattle were ultimately enrolled.

Invitations to participate were distributed through the BDCVS to Provincial Livestock Offices nationwide. Provinces that agreed to participate and reported ongoing or recent LSD outbreaks were included. Eligible farms were required to have at least one animal exhibiting clinical signs consistent with LSD, including skin lesions and enlargement of superficial lymph nodes. Sampled cattle were required to have resided on the participating farm for at least 1 month before sample collection. One or more cattle were purposively selected from each farm according to the availability of eligible animals and feasibility of sampling under field conditions. Farms and cattle that did not meet these eligibility criteria were excluded. If the farm did not have animals showing clinical signs, at least one asymptomatic animal was selected.

A total of 523 samples were collected from 482 beef cattle on 208 farms. These comprised 387 hair samples and 136 skin lesion samples, including nodules, fistulas, sit-fasts, scabs, and scars (Table 1). Hair samples were collected by plucking at least 20 hairs with forceps from the skin over suspected lesions. Skin lesion samples of at least 0.5 × 0.5 cm were obtained by biopsy or collected as necropsy tissue. Samples were placed in appropriate collection containers and transported to the laboratory at 4°C within 24 h of collection. Samples were subsequently stored at −20°C until LSDV detection. Animal-level demographic and epidemiological information, including age, sex, clinical signs, and vaccination history, was recorded.

**Table 1:** Distribution of beef cattle farms, sampled animals, and sample types across 27 provinces of Thailand.

**Province**	**Region**	**Farms, n**	**Animals, n**	**Hair samples, n**	**Skin lesion samples, n**
Chiang Rai	Northern	7	10	9	1
Phetchabun	Northern	5	20	20	–
Phitsanulok	Northern	4	20	20	–
Sukhothai	Northern	5	20	20	–
Ang Thong	Central	12	18	18	–
Bangkok	Central	1	1	1	–
Lop Buri	Central	5	5	2	3
Nonthaburi	Central	4	20	20	–
Phra Nakhon Si Ayutthaya	Central	5	8	8	4
Saraburi	Central	4	13	13	–
Suphan Buri	Central	5	22	21	2
Buri Ram	Northeastern	4	5	3	2
Chaiyaphum	Northeastern	15	30	22	15
Khon Kaen	Northeastern	16	43	13	30
Mukdahan	Northeastern	5	20	17	6
Roi Et	Northeastern	22	22	6	16
Ubon Ratchathani	Northeastern	15	32	17	15
Sa Kaeo	Eastern	5	14	14	8
Kanchanaburi	Western	5	20	18	3
Phetchaburi	Western	5	19	17	12
Prachuap Khiri Khan	Western	2	8	8	–
Ratchaburi	Western	23	39	27	16
Tak	Western	1	20	20	1
Chumphon	Southern	7	20	20	–
Narathiwat	Southern	1	4	4	–
Pattani	Southern	20	20	20	–
Trang	Southern	5	9	9	2
Total		208	482	387	136

A total of 523 samples were obtained from 482 cattle because paired hair and skin lesion samples were collected from 41 animals. – = No sample collected.

### DNA extraction and LSDV detection

All samples were pretreated using an Omni Bead Ruptor 12 bead mill homogenizer (Omni International, Kennesaw, GA, USA). Approximately 0.5 cm³ of each skin lesion sample or 10–20 hairs were mixed with 300 µL of phosphate-buffered saline (PBS; pH 7.4) and homogenized according to the manufacturer's instructions. Viral DNA was subsequently extracted from 200 µL of each pretreated sample using the DNA/RNA GENTi™ 32 Automated Nucleic Acid Extraction System (GeneAll Biotechnology Co., Ltd., Seoul, Republic of Korea) according to the manufacturer's instructions. Extracted DNA was stored at −20°C until analysis.

LSDV was detected using conventional PCR targeting the *RPO30* gene. The forward primer 5′-TCTATGTC TTGATATGTGGTGGTAG-3′ and reverse primer 5′-AGTGATTAGGTGGTGTATTATTTTCC-3′ were used as previously described [16]. The assay was performed according to the validated protocol described by Lamien *et al.* [16], which had a reported analytical detection limit of 250 copies/mL and high analytical specificity. PCR amplification consisted of initial denaturation at 95°C for 15 min, followed by 40 cycles of denaturation at 94°C for 30 s, annealing at 50°C for 30 s, and extension at 72°C for 30 s, followed by a final extension at 72°C for 5 min. PCR products were analyzed by agarose gel electrophoresis, with an expected amplicon size of 172 bp. A DNA sample previously confirmed as LSDV-positive using a validated PCR assay was included as the positive control in each PCR run, and nuclease-free water served as the no-template negative control. DNA extraction and PCR detection were performed at the Center of Excellence for Emerging and Re-emerging Infectious Diseases in Animals (CUEIDAs), Faculty of Veterinary Science, Chulalongkorn University, Bangkok, Thailand.

### Characterization of LSDV by *RPO30* and *GPCR* gene sequencing

For molecular characterization, the *RPO30* and *GPCR* genes of LSDV were amplified by PCR using specific primers with minor modifications to a previously described method [5]. Seventeen LSDV-positive samples were selected for sequencing based on DNA quality, geographic location, and collection date. DNA concentration and purity were assessed using a NanoDrop spectrophotometer, with a concentration >20 ng/µL and an A260/A280 ratio >1.80 used as the stated quality criteria.

Each 50-µL PCR mixture contained 5 µL of viral DNA, 0.4 µM each of the forward and reverse primers, 1× HotStarTaq Plus Master Mix (Qiagen, Valencia, CA, USA), and nuclease-free water to a final volume of 50 µL. Amplification consisted of initial denaturation at 95°C for 15 min, followed by 40 cycles of denaturation at 94°C for 30 s, annealing at 45°C for 30 s, and extension at 72°C for 90 s, followed by a final extension at 72°C for 15 min. PCR products were separated on a 1.5% agarose gel prepared in 0.5× Tris-borate-EDTA (TBE) buffer. A 100-bp GeneRuler DNA ladder (Thermo Scientific) was used to verify product sizes. The expected amplicon sizes were 606 bp for *RPO30* and 1,134 bp for *GPCR*.

Following electrophoresis, PCR products were purified using the NucleoSpin Gel and PCR Clean-up Kit (Macherey-Nagel, Landsmeer, The Netherlands) according to the manufacturer's instructions and subjected to bidirectional Sanger sequencing by U2Bio (Bangkok, Thailand). Forward and reverse chromatograms were manually inspected, edited, validated, and assembled using SeqMan version 5.03 (DNASTAR Inc., Madison, WI, USA) to generate consensus sequences. Ambiguous nucleotide calls were resolved by comparison of the corresponding forward and reverse reads. Consensus sequences were compared with sequences available in the National Center for Biotechnology Information database using the Basic Local Alignment Search Tool (BLAST) to confirm sequence identity and were subsequently exported in FASTA format for further analysis. The nucleotide sequences generated in this study were deposited in GenBank under accession numbers PZ284682–PZ284715.

### Phylogenetic and genetic analyses of LSDV

Phylogenetic analyses were performed by comparing the nucleotide sequences of the *RPO30* and *GPCR* genes generated in this study with reference sequences of members of the genus *Capripoxvirus*, including goatpox virus (GTPV), sheeppox virus (SPPV), and LSDV strains representing different clades and geographic origins in Africa, Asia, and Europe available in GenBank. Phylogenetic trees based on the *RPO30* and *GPCR* sequences were constructed using MEGA version 11.0 with the neighbor-joining method, Kimura two-parameter model, and 1,000 bootstrap replicates. The Kimura two-parameter model was chosen *a priori*.

For genetic analysis, the *GPCR* nucleotide sequences of Thai LSDVs were aligned with reference LSDV sequences representing clades 1.1, 1.2, 2.1–2.4, and 2.5. Multiple sequence alignment and pairwise nucleotide identity analyses were performed using MEGA version 11.0.

### Statistical analysis

Associations between LSDV positivity and animal age, sex, clinical status, and vaccination history were evaluated using individual cattle as the unit of analysis. The statistical model uses individual cattle as the unit of analysis, while farm-level clustering was not considered. Univariable logistic regression and chi-square tests were initially performed. Variables with p < 0.20 in the initial analysis were considered for inclusion in the multivariable logistic regression model. Multivariable logistic regression was subsequently performed to identify factors independently associated with LSDV positivity, and adjusted odds ratio (aOR) with 95% confidence intervals (CIs) were calculated. Statistical significance was established at p < 0.05. Statistical analyses were performed using R software version 4.2 and IBM SPSS Statistics version 29.0.1.0.

## RESULTS

### Epidemiological findings of LSD outbreaks in Thailand in 2022

Farm characteristics and management practices were assessed using the epidemiological questionnaire, and the findings are presented in Table 2. Among the 208 beef cattle farms surveyed, 129 (62.02%) were classified as small farms (1–10 animals/farm), 54 (25.96%) as medium farms (>10 to <30 animals/farm), and 25 (12.02%) as large farms (≥30–277 animals/farm).

Regarding management actions taken for cattle suspected of LSDV infection (multiple responses were permitted), 200/208 (96.15%) farms reported treating affected cattle with antibiotics. In addition, 13/208 (6.25%) farms reported treatment combined with selling cattle for slaughter, and 8/208 (3.85%) reported treatment combined with selling cattle to another farm. One farm reported all three practices, whereas 8/208 (3.85%) farms reported taking no action.

For insect control, 58/208 (27.88%) farms reported no control measures, whereas 50/208 (24.04%) used nets only, 22/208 (10.58%) used insecticides only, 26/208 (12.50%) used smoke only, and 1/208 (0.48%) used native herbs only. Multiple insect control methods were used concurrently on 50/208 (24.04%) farms. Regarding farm cleaning, 181/208 (87.02%) farms were reported as always being cleaned, whereas 27/208 (12.98%) were reported as never being cleaned. Soil flooring was used on 169/208 (81.25%) farms, concrete flooring on 21/208 (10.10%), and a combination of soil and concrete flooring on 18/208 (8.65%). New animals had been purchased by 24/208 (11.54%) farms, whereas 184/208 (88.46%) reported no recent animal purchases. In addition, 89/208 (42.79%) farms allowed contact between their cattle and cattle from outside the farm (Table 2).

**Table 2:** Farm characteristics and management practices during lumpy skin disease outbreaks in Thailand in 2022.

**Category**	**Variable**	**Farms, n (N = 208)**	**Percentage**
Farm size	Small (1–10 animals)	129	62.02
	Medium (>10 to <30 animals)	54	25.96
	Large (≥30–277 animals)	25	12.02
Treatment actionsᵃ	Treated with antibiotics	200	96.15
	Treated and sold for slaughter	13	6.25
	Treated and sold to another farm	8	3.85
	Performed all three actions	1	0.48
	Took no action	8	3.85
Insect control	No measures taken	58	27.88
	Nets only	50	24.04
	Insecticides only	22	10.58
	Smoke only	26	12.50
	Herbs only	1	0.48
	Electric insect traps only	1	0.48
	Multiple methods	50	24.04
	Nets and insecticides	24	11.54
	Nets and smoke	7	3.37
	Nets and electric insect traps	1	0.48
	Nets, smoke, and insecticides	4	1.92
	Insecticides and smoke	12	5.77
	Insecticides and herbs	2	0.96
Farm cleaning	Always clean	181	87.02
	Never clean	27	12.98
Flooring type	Soil flooring	169	81.25
	Concrete flooring	21	10.10
	Both types	18	8.65
Purchasing new animals	Yes	24	11.54
	No	184	88.46
Contact with outside cattle	Yes	89	42.79
	No	119	57.21

ᵃTreatment-action categories were not mutually exclusive; individual farms could report more than one action. Antibiotics are used when secondary bacterial infections are suspected or confirmed.

^b ^Farm cleaning: Tap water and disinfectant were used for farm cleaning.

### Occurrence and factors associated with LSDV positivity

Among the 482 cattle sampled from 208 farms across 27 provinces, 405 (84.02%) exhibited clinical signs at the time of sampling, whereas 77 (15.98%) showed no apparent clinical signs. Clinical manifestations included skin nodules with or without sit-fasts and scabs, fistulas, scars, nasal and/or ocular discharge, edema, and systemic signs (Figure 2). The most frequently recorded clinical manifestations were skin nodules with scabs (80.95%), followed by scars (25.56%), systemic signs (6.27%), fistulas (3.26%), edema (3.26%), and nasal and/or ocular discharge (2.76%). Individual cattle could exhibit more than one clinical manifestation. The reported duration until recovery from clinical signs ranged from 1 to 6 months, with an average of 2 months (Supplementary Table 1).

**Figure 2:**
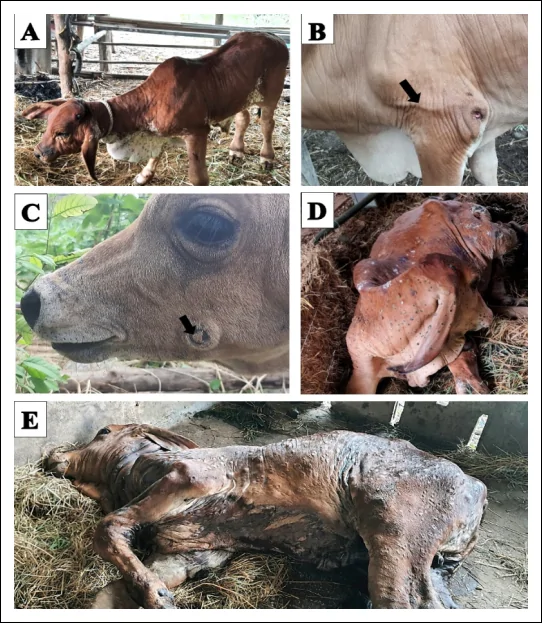
Clinical manifestations of LSD in cattle. (A) Nodules, subcutaneous edema, and ocular and nasal discharge; (B) fistula formation; (C) nodule with a sit-fast lesion; (D) scab and scar formation; and (E) lateral recumbency accompanied by fever, dyspnea, and nodular lesions. LSD = Lumpy skin disease.

Overall, 358/482 (74.27%) cattle tested positive for LSDV. Among cattle exhibiting clinical signs, 304/405 (75.06%) were LSDV-positive, compared with 54/77 (70.13%) cattle without apparent clinical signs. According to age group, LSDV positivity was highest among weaners (>6 to <12 months), at 82.35% (70/85), followed by calves (<6 months), at 81.20% (108/133), and adults (≥12 months), at 68.18% (180/264). Positivity was 75.65% (146/193) among males and 73.36% (212/289) among females. According to vaccination status, LSDV positivity was 79.30% (226/285) among unvaccinated cattle, 65.70% (113/172) among vaccinated cattle, and 76.00% (19/25) among cattle with unknown vaccination status.

Multivariable logistic regression identified age and vaccination status as factors significantly associated with LSDV positivity (p < 0.05; Table 3). Compared with adult cattle (≥12 months), weaners (>6 to <12 months) had 1.89-fold higher adjusted odds of LSDV positivity (aOR = 1.89; 95% CI: 1.02–3.69; p = 0.049), whereas calves (≤6 months) had 1.73-fold higher aOR (1.73; 95% CI: 1.01–3.01; p = 0.048). Vaccinated cattle had lower adjusted odds of LSDV positivity than unvaccinated cattle (aOR = 0.62; 95% CI: 0.39–0.98; p = 0.041).

At the sample level, 270/387 (69.77%) hair samples and 125/136 (91.91%) skin lesion samples tested positive for LSDV. The chi-square test showed a significant association between sample type and LSDV detection (χ² = 25.51, p < 0.05). Univariable logistic regression further showed that skin lesion samples had higher odds of testing positive than hair samples (OR = 4.92; 95% CI: 2.56–9.47; p < 0.05) (Table 3).

### *RPO30* and *GPCR* gene sequences and phylogenetic analysis

The *RPO30* and *GPCR* sequences generated from 17 Thai LSDV-positive samples were compared with reference sequences representing members of the genus *Capripoxvirus* from different geographic regions and collection periods. Pairwise sequence analysis showed 100% nucleotide identity among all 17 Thai LSDVs for both the *RPO30* and *GPCR* genes.

**Table 3:** Chi-square and logistic regression analyses of factors associated with LSDV positivity in beef cattle.

**Variable**	**Tested, n**	**LSDV-positive, n (%)**	**χ² (** **df** **)**	**p** **-value**	**Crude OR**	**95% CI**	**p-value**	**aOR**	**95% CI**	**p-value**
Age			11.37 (2)	0.003						
Adult (≥12 months)	264	180 (68.18)			Ref.			Ref.		
Weaner (>6 to <12 months)	85	70 (82.35)			2.18	1.18–4.03	0.013	1.89	1.02–3.69	0.049
Calf (≤6 months)	133	108 (81.20)			2.02	1.21–3.34	0.007	1.73	1.01–3.01	0.048
Sex			0.21 (1)	0.647						
Male	193	146 (75.65)			Ref.			–	–	–
Female	289	212 (73.36)			0.89	0.58–1.34	0.573	–	–	–
Clinical status			0.58 (1)	0.441						
Without clinical signs	77	54 (70.13)			Ref.			–	–	–
Clinical signs	405	304 (75.06)			1.28	0.75–2.19	0.365	–	–	–
Vaccination status			10.42 (2)	0.005						
Unvaccinated	285	226 (79.30)			Ref.			Ref.		
Vaccinated	172	113 (65.70)			0.50	0.33–0.77	0.001	0.62	0.39–0.98	0.041
Unknown	25	19 (76.00)			0.83	0.32–2.16	0.698	1.11	0.45–2.23	0.837
Sample typeᵃ			25.51 (1)	<0.001						
Hair	387	270 (69.77)			Ref.			–	–	–
Skin lesion	136	125 (91.91)			4.92	2.56–9.47	<0.001	–	–	–

ᵃPaired hair and skin lesion samples were collected from 41 cattle. A total of 523 samples were analyzed, of which 395 (75.53%) were LSDV-positive. Variables considered for inclusion in the multivariable logistic regression model were age and vaccination status. aOR = Adjusted odds ratio; CI = Confidence interval; LSDV = Lumpy skin disease virus; OR = Odds ratio; Ref. = Reference category; – = Not included in the multivariable model; the variable with p<0.20 is included in the multivariable analysis. Statistical significance was set at p < 0.05.

For the *RPO30* gene, the Thai LSDVs shared 100% nucleotide identity with representative field strains from China, Indonesia, Hong Kong, Vietnam, and previously reported Thai LSDVs. In comparison, they shared 99.67% nucleotide identity with the classical Neethling and KSGP-O240 vaccine strains. For the *GPCR* gene, the Thai LSDVs shared 100% nucleotide identity with representative strains from Indonesia, Hong Kong, Thailand, and Vietnam, 99.91% identity with the representative Chinese strain, and 98.83% identity with the classical Neethling and KSGP-O240 vaccine strains.

Phylogenetic analyses based on the *RPO30* and *GPCR* genes separated the analyzed LSDV reference strains into two major clades, clades 1 and 2, with additional subclades within each major group. In the *RPO30*-based phylogenetic tree, the Thai LSDVs clustered within clade 2.5 together with field strains from China (Xinjiang/19; OP508345), Hong Kong (Hong Kong/20; MW732649), Indonesia (A04224595/22; PQ037207), Thailand (YST/21; OM033705), and Vietnam (Bang-Thanh/20; MZ577076) (Figure 3). Similarly, phylogenetic analysis of the *GPCR* gene placed the Thai LSDVs within clade 2.5 together with LSDVs reported from China, Hong Kong, Indonesia, Thailand, and Vietnam (Figure 4).

Multiple sequence alignment of the *GPCR* gene showed that the Thai LSDV sequences were identical and contained a 12-nucleotide insertion (AGCACAATTCTC) at positions 94–105. A corresponding 12-nucleotide insertion was also present in LSDVs belonging to clade 1.1, some clade 1.2 viruses reported from Bangladesh and Kenya, and recombinant LSDV strains belonging to clades 2.1–2.5. In contrast, this insertion was absent from the analyzed wild-type subclade 1.2 LSDVs originating from Africa, the Middle East, and Europe. Among the analyzed subclade 1.2 viruses, the insertion was present in subclade 1.2.2 strains from India but absent from subclade 1.2.1 strains from Africa, the Middle East, Europe, Russia, and Kazakhstan (Table 4 and Figure 5).

## DISCUSSION

### Epidemiological characteristics and factors associated with LSDV positivity

This study provides epidemiological and molecular information on LSDV circulation in beef cattle in Thailand during 2022, approximately 1 year after the first reported outbreak in the country. The overall LSDV positivity among sampled cattle, determined by conventional PCR targeting the *RPO30* gene, was 74.27%. Skin lesion samples yielded a substantially higher positivity rate (91.91%) than hair samples (69.77%). Comparable positivity rates in skin lesion samples have been reported previously in Thailand and Bangladesh, at 92.79% and 83.89%, respectively [17, 18], whereas another study in Thailand reported an overall LSDV positivity rate of 70.13% [11]. Differences among studies may reflect variation in study populations, outbreak stage, sample type, sampling strategy, and diagnostic approaches; however, these factors cannot be evaluated directly from the present dataset.

Age and vaccination status were significantly associated with LSDV positivity. Weaners (>6 to <12 months) and calves (≤6 months) had higher adjusted odds of LSDV positivity than adult cattle (≥12 months). Similar age-related patterns have been reported previously [19]. Although differences in immune status may contribute to greater susceptibility among younger animals, the present study did not directly assess immune competence, maternally derived antibodies, or individual immunological status. Therefore, the biological mechanisms underlying the observed age association cannot be established from these data.

**Figure 3:**
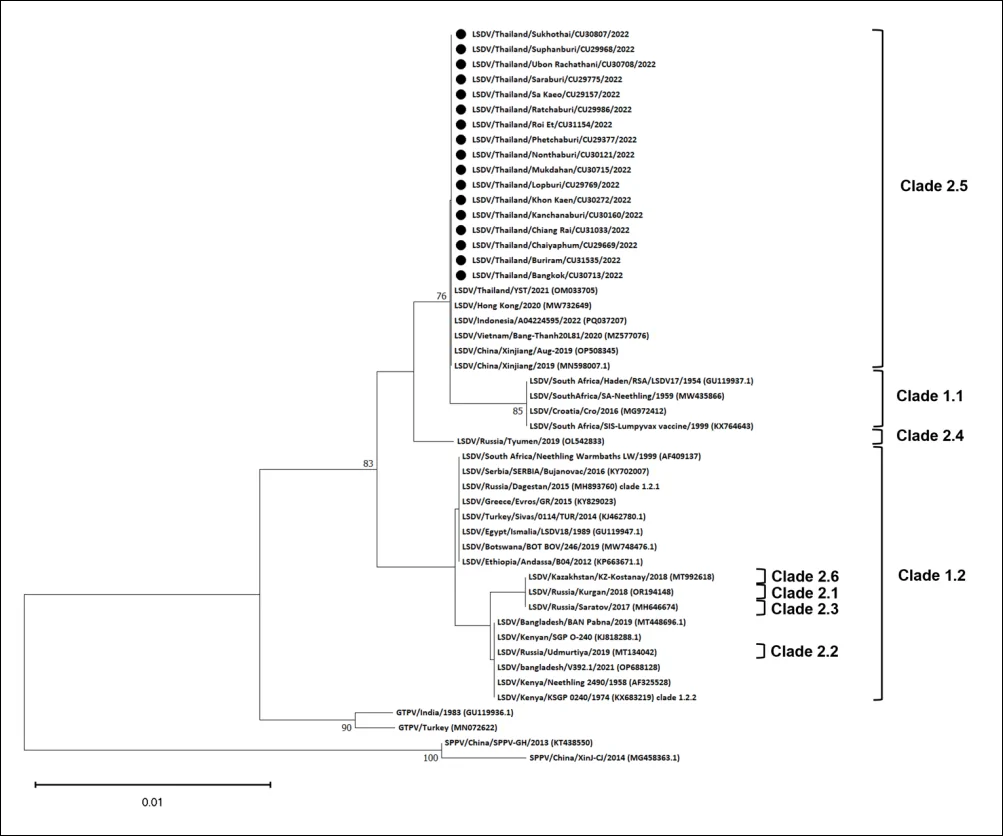
Neighbor-joining phylogenetic tree based on the *RPO30* gene sequences of Thai LSDVs and representative *Capripoxvirus* reference strains. The tree was constructed using MEGA version 11 with the neighbor-joining method, Kimura two-parameter model, and 1,000 bootstrap replicates. Bootstrap support values are shown at the nodes. GTPV and SPPV sequences were used as outgroups to root the tree. The scale bar indicates the number of nucleotide substitutions per site. Black circles indicate Thai LSDVs identified in this study. GTPV = Goatpox virus; LSDV = Lumpy skin disease virus; SPPV = Sheeppox virus.

Vaccinated cattle had lower adjusted odds of LSDV positivity than unvaccinated cattle (aOR = 0.62), supporting an association between vaccination and reduced likelihood of LSDV detection. This finding reinforces the importance of vaccination as a component of LSD prevention and control. Nevertheless, because the study was cross-sectional and information on vaccine type, number of doses, timing of vaccination relative to exposure, and post-vaccination immune response was not incorporated into the reported analysis, vaccine effectiveness should not be inferred directly from the observed odds ratio.

### Diagnostic value of skin lesion and hair samples

Skin lesion samples demonstrated a higher LSDV positivity rate than hair samples, with 91.91% of skin samples and 69.77% of hair samples testing positive. Univariable analysis further showed that skin lesion samples had higher odds of LSDV detection than hair samples. This finding is consistent with the well-established suitability of skin lesions for molecular detection of LSDV during clinical disease [15]. However, hair samples also yielded a substantial proportion of positive results, indicating their potential utility as an alternative specimen type under field conditions.

**Figure 4:**
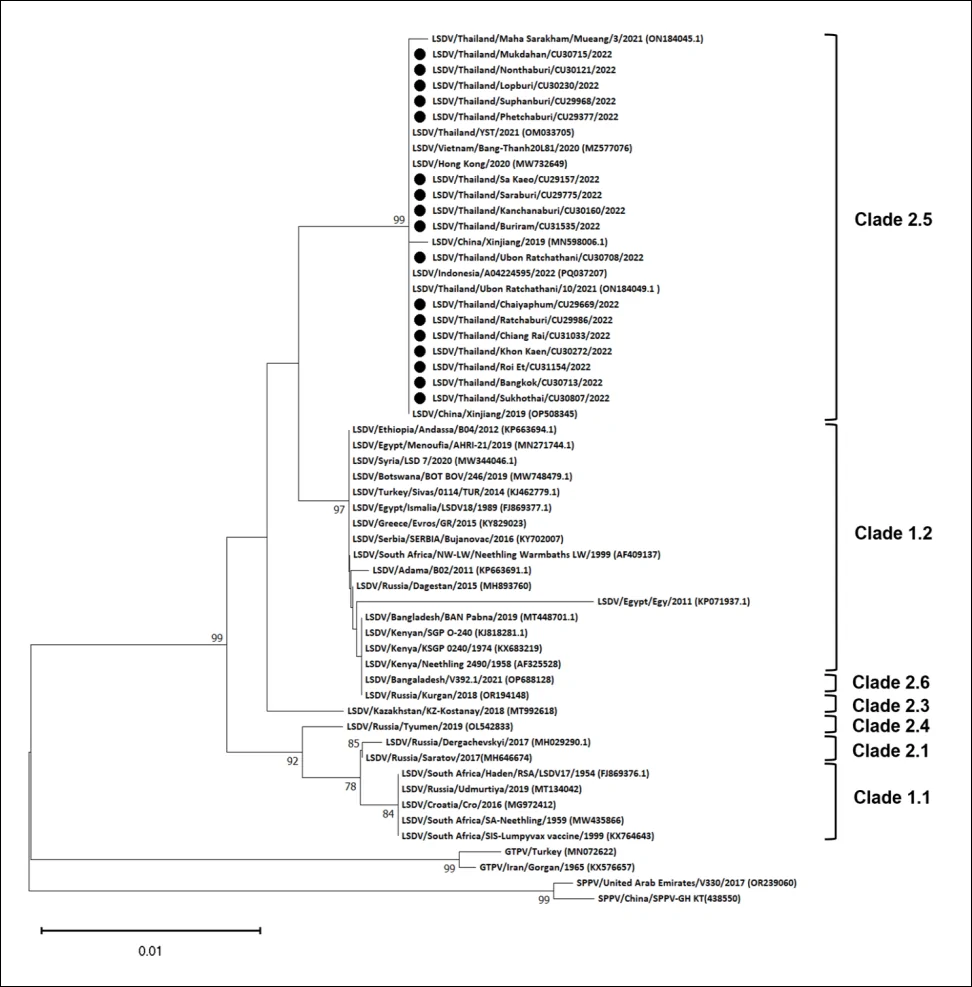
Neighbor-joining phylogenetic tree based on the *GPCR* gene sequences of Thai LSDVs and representative *Capripoxvirus* reference strains. The tree was constructed using MEGA version 11 with the neighbor-joining method, Kimura two-parameter model, and 1,000 bootstrap replicates. Bootstrap support values are shown at the nodes. GTPV and SPPV sequences were used as outgroups to root the tree. The scale bar indicates the number of nucleotide substitutions per site. Black circles indicate Thai LSDVs identified in this study. GPCR = G protein-coupled receptor; GTPV = Goatpox virus; LSDV = Lumpy skin disease virus; SPPV = Sheeppox virus.

**Table 4:** Characteristics of Thai LSDVs analyzed in this study and reference LSDV strains used for GPCR and RPO30 sequence analyses.

**Virus**	**Date**	**Age**	**Province/** **country**	Clade*	***GPCR*** ** insertion (positions 94–105)**	**GenBank accession no. ** **(** ***GPCR*** **, ** ***RPO30*** **, or WGS)**
Reference viruses						
LSDV/South Africa/Haden/RSA/LSDV17/1954	1954	N/A	South Africa	1.1	12-bp insertion	FJ869376.1, GU119937.1
LSD/South Africa/Neethling (Vaccine)	1959	N/A	South Africa	1.1	12-bp insertion	AF409138.1 (WGS)
LSDV/Egypt/Egy/2011	2011	N/A	Egypt	1.2	No insertion	KP071937.1
LSDV/Ethiopia/Andassa/B04/2012	Oct 2012	N/A	Ethiopia	1.2	No insertion	KP663694.1, KP663671.1
LSDV/Egypt/Menoufia-AHRI-21/2019	2019	N/A	Egypt	1.2	No insertion	MN271744.1
LSDV/Syria/LSD-7/2020	Jul 2020	N/A	Syria	1.2	No insertion	MW344046.1
LSDV/Botswana/BOT_BOV/246/2019	2019	N/A	Botswana	1.2	No insertion	MW748479.1, MW748476.1
LSDV/Turkey/Sivas/0114/2014	Jan 2014	N/A	Turkey	1.2	No insertion	KJ462779.1, KJ462780.1
LSDV/Egypt/Ismailia/LSDV18/1989	1989	N/A	Egypt	1.2	No insertion	FJ869377.1, GU119947.1
LSDV/Bangladesh/BAN_Pabna/2019	Sep 2019	N/A	Bangladesh	1.2	12-bp insertion	MT448701.1, MT448696.1
LSDV/Kenya/KSGP-O240 (Vaccine)	1974	N/A	Kenya	1.2	12-bp insertion	KX683291.1 (WGS)
LSDV/Kenya/Neethling-2490	1958	N/A	Kenya	1.2	12-bp insertion	AF325528.1 (WGS)
LSDV/Russia/Dergachevskyi/2017	Jun 2017	N/A	Russia	2.1	12-bp insertion	MH029290.1
LSDV/Russia/Saratov/2017	2017	N/A	Russia	2.1	12-bp insertion	MH646674.1 (WGS)
LSDV/Russia/Udmurtiya/2019	Mar 2019	N/A	Russia	2.2	12-bp insertion	MT134042.1 (WGS)
LSDV/Kazakhstan/KZ-Kostanay/2018	Sep 2018	N/A	Kazakhstan	2.3	12-bp insertion	MT992618.1 (WGS)
LSDV/Russia/Tyumen/2019	2019	N/A	Russia	2.4	12-bp insertion	OL542833.1 (WGS)
LSDV/China/GD01/2020	2020	N/A	China	2.5	12-bp insertion	MW355944.1 (WGS)
LSDV/China/Xinjiang/2019	Oct 2019	N/A	China	2.5	12-bp insertion	MN598006.1, MN598007.1
LSDV/Vietnam/Bang-Thanh/20L81/2020	Nov 2020	N/A	Vietnam	2.5	12-bp insertion	MZ577076.1 (WGS)
LSDV/Indonesia/A04224595/2022	Oct 2022	N/A	Indonesia	2.5	12-bp insertion	PQ037207.1 (WGS)
LSDV/Hong Kong/2020	Dec 2020	N/A	Hong Kong	2.5	12-bp insertion	MW732649.1 (WGS)
LSDV/Thailand/YST/2021	Apr 2021	N/A	Thailand	2.5	12-bp insertion	OM033705.1 (WGS)
LSDV/Thailand/Maha Sarakham/3/2021	Nov 2021	N/A	Thailand	2.5	12-bp insertion	ON184045.1
LSDV/Thailand/Ubon Ratchathani/10/2021	Jul 2021	N/A	Thailand	2.5	12-bp insertion	ON184049.1
Viruses characterized in this study						
CU29157	Jul 2022	**≥**12 months	Sa Kaeo, Thailand	2.5	12-bp insertion	PZ284682(GPCR), PZ284683(RPO30)
CU29377	Aug 2022	**≥**12 months	Phetchaburi, Thailand	2.5	12-bp insertion	PZ284684(GPCR), PZ284685(RPO30)
CU29669	Aug 2022	≤6 months	Chaiyaphum, Thailand	2.5	12-bp insertion	PZ284686(GPCR), PZ284687(RPO30)
CU29775	Sep 2022	>6–<12 months	Saraburi, Thailand	2.5	12-bp insertion	PZ284688(GPCR), PZ284689(RPO30)
CU29968	Sep 2022	>6–<12 months	Suphan Buri, Thailand	2.5	12-bp insertion	PZ284690(GPCR), PZ284691(RPO30)
CU29986	Jul 2022	**≥**12 months	Ratchaburi, Thailand	2.5	12-bp insertion	PZ284692(GPCR), PZ284693(RPO30)
CU30160	Sep 2022	≤6 months	Kanchanaburi, Thailand	2.5	12-bp insertion	PZ284694(GPCR), PZ284695(RPO30)
CU30230	Sep 2022	≤6 months	Lop Buri, Thailand	2.5	12-bp insertion	PZ284696(GPCR), PZ284697(RPO30)
CU31033	Nov 2022	>6–<12 months	Chiang Rai, Thailand	2.5	12-bp insertion	PZ284698(GPCR), PZ284699(RPO30)
CU31535	Dec 2022	≤6 months	Buri Ram, Thailand	2.5	12-bp insertion	PZ284700(GPCR), PZ284701(RPO30)
CU30121	Sep 2022	**≥**12 months	Nonthaburi, Thailand	2.5	12-bp insertion	PZ284702(GPCR), PZ284703(RPO30)
CU30272	Sep 2022	≤6 months	Khon Kaen, Thailand	2.5	12-bp insertion	PZ284704(GPCR), PZ284705(RPO30)
CU30708	Oct 2022	≤6 months	Ubon Ratchathani, Thailand	2.5	12-bp insertion	PZ284706(GPCR), PZ284707(RPO30)
CU30715	Sep 2022	≤6 months	Mukdahan, Thailand	2.5	12-bp insertion	PZ284708(GPCR), PZ284709(RPO30)
CU31154	Sep 2022	>6–<12 months	Roi Et, Thailand	2.5	12-bp insertion	PZ284710(GPCR), PZ284711(RPO30)
CU30713	Nov 2022	≤6 months	Bangkok, Thailand	2.5	12-bp insertion	PZ284712(GPCR), PZ284713(RPO30)
CU30807	Nov 2022	**≥**12 months	Sukhothai, Thailand	2.5	12-bp insertion	PZ284714(GPCR), PZ284715(RPO30)

*GPCR* = G protein-coupled receptor gene; LSDV = Lumpy skin disease virus; N/A = Not applicable; *RPO30* = RNA polymerase 30-kDa subunit gene; WGS = Whole-genome sequence. The 12-bp *GPCR* insertion corresponds to positions 94–105 in the alignment used in this study.

*Clade classification: LSDV strains have been classified using whole-genome sequences and commonly analyzed genetic markers, including *GPCR* and *RPO30* [3].

Hair samples were collected by plucking hairs to include the roots, where detectable viral material may be present [15]. Compared with skin biopsy, hair collection is less invasive and may be more practical for repeated or large-scale field sampling. Importantly, LSDV was also detected among cattle without apparent clinical signs, suggesting that hair or other minimally invasive samples may have value when lesion material is unavailable. Nevertheless, the present study did not directly evaluate diagnostic sensitivity, specificity, agreement between paired hair and skin samples, or animal-level paired performance. Therefore, the findings support the potential practical utility of hair samples but do not establish equivalence with skin lesion sampling.

**Figure 5:**
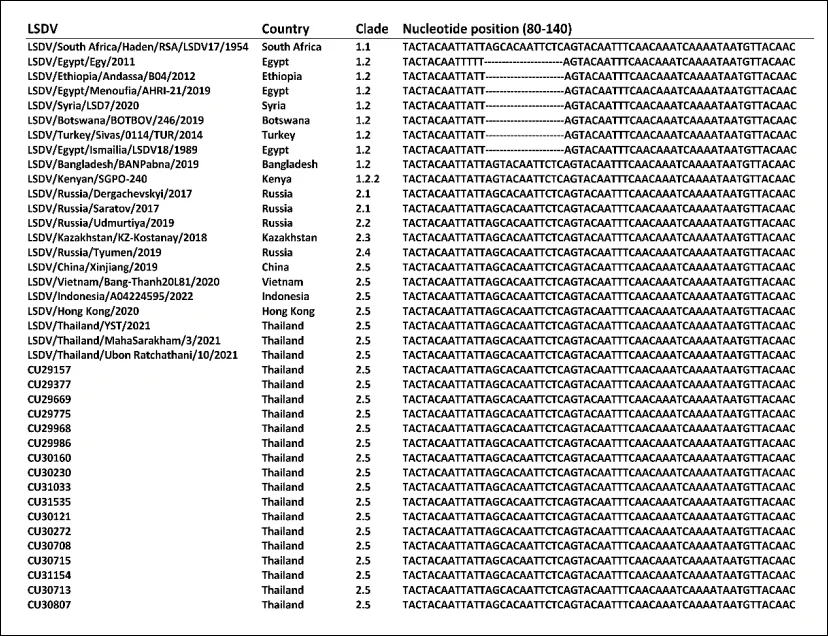
Multiple sequence alignment of the *GPCR* gene showing the 12-bp insertion at nucleotide positions 94–105 among Thai LSDVs and representative reference strains. GPCR = G protein-coupled receptor; LSDV = Lumpy skin disease virus. Reference sequence for multiple sequence alignment: LSDV/South Africa/Haden/RSA/LSDV17/1954.

### Farm management practices during LSD outbreaks

Several farm management practices observed during the outbreaks warrant attention. Approximately 28% of farms reported no insect control measures, despite the recognized role of blood-feeding arthropods in LSDV transmission. Strengthening vector control practices may therefore be relevant to integrated outbreak management, particularly during periods of high vector abundance.

Antibiotic administration was reported on approximately 96% of surveyed farms. Because antibiotics do not act against LSDV, their use would generally be relevant only when secondary bacterial infections are suspected or confirmed. The present study did not record antibiotic classes, indications, dosages, treatment duration, withdrawal-period compliance, or veterinary prescription practices. Therefore, inappropriate antimicrobial use or withdrawal-period violations cannot be concluded from these data. Nevertheless, the high frequency of reported antibiotic administration highlights the need for further investigation of antimicrobial use practices during LSD outbreaks and for appropriate veterinary guidance regarding secondary bacterial complications and food-animal withdrawal requirements [4, 19].

### Molecular characteristics and phylogenetic placement of Thai LSDVs

LSDV strains have been classified into major phylogenetic clades using whole-genome sequences and commonly analyzed genetic markers, including *GPCR* and *RPO30*. Clade 1 includes clades 1.1 and 1.2. Clade 1.1, corresponding to Neethling-type viruses, includes classical vaccine-associated strains reported predominantly from sub-Saharan Africa. Clade 1.2, including Kenya/KSGP-type viruses, comprises field strains reported from Africa, Europe, and Asia and has been further divided into subclades 1.2.1, 1.2.2, and 1.2.3. Subclade 1.2.1 has been associated with outbreaks in the Middle East and Europe, including Balkan and Russian wild-type strains; subclade 1.2.2 includes Kenya-like strains reported in India and Bangladesh; and subclade 1.2.3 includes recently characterized strains reported from West and Central Africa. In contrast, clade 2 comprises recombinant viruses containing genomic regions derived from wild-type and vaccine-like LSDVs. Clades 2.1–2.4 have been reported mainly from Russia and Kazakhstan, whereas clade 2.5 has been reported in East and Southeast Asia, including China, Thailand, and Vietnam [3, 17].

Phylogenetic analyses based on the *RPO30* and *GPCR* genes are commonly used for molecular characterization and evolutionary investigation of LSDV [2, 5, 20]. In the present study, all 17 characterized Thai LSDVs clustered within clade 2.5 based on both gene regions, together with viruses reported from China, Hong Kong, Indonesia, Thailand, and Vietnam [21]. The analyzed Thai sequences also showed complete nucleotide identity with one another in both *RPO30* and *GPCR*, indicating high sequence conservation across these two loci among the sampled viruses. However, because only two gene regions were analyzed, these findings should not be interpreted as evidence of genome-wide genetic stability.

The clustering of Thai LSDVs with clade 2.5 strains reported elsewhere in East and Southeast Asia demonstrates close regional genetic relatedness. This finding is consistent with previous reports describing circulation of recombinant clade 2.5 LSDVs in China, Indonesia, Vietnam, and Thailand [11, 17, 21]. In contrast, LSDVs reported from Bangladesh, India, and Myanmar have been classified within clade 1.2 [4]. These contrasting phylogenetic patterns indicate that genetically distinct LSDV lineages have circulated in different parts of Asia. However, phylogenetic similarity alone cannot determine the direction, timing, or route of virus movement between countries.

### Significance of the 12-nucleotide *GPCR* insertion

Multiple sequence alignment of the *GPCR* gene demonstrated a 12-nucleotide insertion at positions 94–105 in the Thai LSDVs characterized in this study. The 12-nucleotide insertion was observed in recombinant LSDV lineages and in some non-recombinant strains (Table 4 and Figure 5). Therefore, the insertion represents a useful comparative molecular feature but should not be considered uniquely specific to clade 2.5.

In the present analysis, the insertion was also observed in clade 1.1, some clade 1.2 viruses, and recombinant clades 2.1–2.5, whereas it was absent from several wild-type subclade 1.2 strains from Africa, the Middle East, and Europe as observed in this study (Figure 5). Accordingly, this sequence feature may contribute to genetic characterization and phylogenetic discrimination when interpreted together with additional sequence information. Its biological significance, including any effect on viral phenotype, vaccine performance, or molecular diagnostic assays, remains uncertain. The potential value of this region for the development of lineage-oriented molecular assays would therefore require dedicated analytical and experimental validation.

### Strengths, limitations, and implications

A major strength of this study was the broad geographic coverage, encompassing 482 cattle from 208 farms across 27 provinces of Thailand, together with the integration of epidemiological assessment, PCR detection, and molecular characterization of representative LSDV-positive samples. The study also generated *RPO30* and *GPCR* sequence data from multiple geographic regions, contributing additional information on the distribution of clade 2.5 LSDVs in Thailand during the 2022 outbreaks. In addition, the identification of age and vaccination status as factors associated with LSDV positivity provides epidemiological information relevant to the prioritization of disease control measures.

Several limitations should be considered when interpreting the findings. First, the cross-sectional design provides a snapshot of LSDV circulation during the study period and does not permit assessment of temporal infection dynamics or causal relationships between explanatory factors and LSDV positivity. Second, provinces and farms were enrolled purposively, with participating farms selected from areas with ongoing or recent suspected LSD outbreaks. Consequently, selection bias is possible, and the observed positivity proportion should not be interpreted as national prevalence. Third, cattle were clustered within farms, and the reported statistical analysis did not indicate whether farm-level clustering was accounted for. Fourth, 523 specimens were obtained from 482 cattle, indicating that some animals contributed more than one sample; therefore, the sample level comparison between hair and skin lesions may include correlated observations. Fifth, molecular characterization was limited to 17 representative LSDV-positive samples and two gene regions, *RPO30* and *GPCR*. Virus isolation and whole-genome sequencing were not performed, limiting inference regarding genome-wide diversity, recombination patterns, and fine-scale transmission relationships.

Future studies should incorporate probability-based or clearly stratified sampling where feasible, longitudinal follow-up of affected farms, and statistical approaches that account for clustering of animals within farms. Direct paired comparisons of hair and skin lesion samples would help determine their relative diagnostic performance. Broader molecular characterization using whole-genome sequencing of viruses from multiple regions and time points would provide greater resolution for evaluating viral diversity, recombinant structure, and regional transmission patterns. Additional investigation of vaccination history, including vaccine type, timing, dose schedule, and post-vaccination status, would also strengthen assessment of the relationship between vaccination and LSDV detection.

## CONCLUSION

This study characterized the epidemiological and molecular features of LSDV infection in beef cattle during the 2022 outbreaks in Thailand using data from 482 cattle on 208 farms across 27 provinces. Overall, 74.27% (358/482) of sampled cattle tested positive for LSDV by conventional PCR targeting the *RPO30* gene. LSDV positivity was higher in skin lesion samples (91.91%) than in hair samples (69.77%). Age and vaccination status were significantly associated with LSDV positivity; weaners and calves had higher adjusted odds of infection than adult cattle, whereas vaccinated cattle had lower adjusted odds than unvaccinated cattle. Molecular characterization of 17 representative LSDV-positive samples showed 100% nucleotide identity among the Thai viruses for the analyzed *RPO30* and *GPCR* gene regions. Phylogenetic analyses placed all characterized Thai LSDVs within recombinant clade 2.5 together with strains reported from China and other East and Southeast Asian countries. The analyzed *GPCR* sequences also contained the 12-nucleotide insertion at positions 94–105 observed in several recombinant and some non-recombinant LSDV lineages.

From a practical perspective, these findings support continued vaccination as an important component of LSD control, with particular attention to younger cattle that showed higher odds of LSDV positivity. The substantial detection of LSDV in cattle without apparent clinical signs also emphasizes that surveillance based solely on clinical examination may fail to identify all infected animals. Although skin lesion samples provided the highest positivity, hair samples yielded a substantial proportion of positive results and may represent a useful, less invasive specimen for field surveillance when lesion material is unavailable. The observed absence of insect control measures on a considerable proportion of farms further highlights the importance of strengthening integrated vector control practices, farmer education, and outbreak management measures. Molecular surveillance should also be maintained to monitor the continued circulation and genetic characteristics of clade 2.5 LSDVs and to detect potential changes in circulating lineages.

In conclusion, the integration of epidemiological investigation with molecular characterization provides important evidence on LSDV circulation in Thai beef cattle following the emergence of the disease in the country. The widespread detection of genetically closely related clade 2.5 viruses across geographically separated study areas indicates substantial regional circulation of this lineage, although the direction and routes of transmission cannot be determined from the present data. Continued vaccination, vector control, epidemiological surveillance, animal movement management, and expanded molecular monitoring, preferably incorporating whole-genome sequencing and broader longitudinal sampling, will be important for strengthening LSD prevention and control in Thailand and for improving understanding of LSDV transmission and evolution within the wider Southeast Asian region.

## DATA AVAILABILITY

The data supporting the findings of this study are available in the Supplementary Tables. The Supplementary Tables can be obtained from the corresponding author. The nucleotide sequences generated in this study are publicly available in the GenBank database under accession numbers PZ284682–PZ284715.

## GENERATIVE AI DECLARATION

The authors declare that generative artificial intelligence (AI) tools were used solely to improve language, grammar, and readability during manuscript preparation. All scientific content, data analysis, interpretation of results, and conclusions were developed and verified by the authors. The authors take full responsibility for the accuracy, integrity, and originality of the work presented, and no AI tool was listed as an author.

## AUTHORS’ CONTRIBUTIONS

PS, EMP, and BP: Sample collection. PS, CN, SC, and KC: Virus detection, viral characterization, and phylogenetic analysis. PS: Writing – original draft. KC and AA: Conceptualization, study design, data analysis, writing – original draft, and writing – review and editing. All authors have reviewed and approved the final manuscript.
